# Identification of *GB1*, a gene whose constitutive overexpression increases glycinebetaine content in maize and soybean

**DOI:** 10.1002/pld3.40

**Published:** 2018-02-22

**Authors:** Paolo Castiglioni, Erin Bell, Adrian Lund, Alexander F. Rosenberg, Meghan Galligan, Brendan S. Hinchey, Stanislaus Bauer, Donald E. Nelson, Robert J. Bensen

**Affiliations:** ^1^ Monsanto Company St. Louis MO USA; ^2^ Present address: Agrotech‐Research Woodland CA USA; ^3^ Present address: Syngenta Crop Protection Glyndon MN USA; ^4^ Present address: University of Alabama at Birmingham Birmingham AL USA; ^5^ Present address: Pfizer Inc. Groton CT USA; ^6^ Present address: Indigo Agriculture, Research Triangle Park NC USA; ^7^ Present address: Syngenta Seeds Stanton MN USA

**Keywords:** GB1, glycinebetaine, stress tolerance, transgenic

## Abstract

Efforts to increase glycinebetaine (GB) levels in plants have been pursued as an approach to improving plant performance under stress conditions. To date, the impact of engineered levels of GB has been limited by metabolic constraints that restrict the achieved increases. We report the identification of a novel gene, *GB1,* that is differentially expressed in high and low GB accumulating maize genotypes. The predicted GB1 protein shows 60% identity to a putative C‐4 sterol methyl oxidase from rice. Overexpression of *GB1* in maize and soybean led to dramatically higher leaf GB content in most of the transgenic lines compared to wild‐type. These results suggest that the GB1 protein is an important component of the biochemical pathways controlling GB accumulation in plants.

## 1 INTRODUCTION

Agricultural productivity is limited by adverse conditions, such as drought, salinity, chilling, freezing, and heat, that impair plant physiological and biochemical processes (Boyer, 1982; Wang, Vinocur, & Altman, 2003). Plants adopt different strategies to tolerate such conditions, including accumulation of compatible solutes (Bohnert, Nelson, & Jensen, 1995). One of the most common and better characterized of these solutes is glycinebetaine (GB). GB occurs widely in nature and many organisms, including bacteria and plants, accumulate GB in response to stress. Moreover, in some plant species, exogenous application of GB has reportedly improved growth or survival under various stress conditions (Allard et al., 1998; Chen, Li, & Chen, 2000; Harinasut et al., 1996; Itai & Paleg, 1982; Makela, Kontturi, Pehu, & Somersalo, 1999; Malekzadeh, 2015).

Two distinct GB biosynthetic pathways are known: a widely occurring choline oxidation pathway common to plants and bacteria and a bacterial specific glycine methylation pathway (Nyyssola, Kerovou, Kaukinen, von Weyrman, & Reinikainen, 2000). For the choline oxidation pathway, different organisms have evolved distinct enzymes to support the conversion of choline into GB via a betaine aldehyde intermediate. In *Escherichia coli*, choline is sequentially oxidized by choline dehydrogenase (CDH) (Styrvold et al., 1986) and betaine aldehyde dehydrogenase (BADH) (Boyd et al., 1991). In *Arthrobacter globiformis,* the choline oxidase enzyme (COX) has been identified as the enzyme responsible for both oxidation steps (Ikuta, Imamura, Misaki, & Horiuti, 1977; Rozwadowski, Khachatourians, & Selvaraj, 1991), while in *Bacillus subtilis*, the *GbsA* and *GbsB* gene products are involved in GB biosynthesis (Boch, Kempf, Schmid, & Bremer, 1996). In plants, choline is oxidized to betaine aldehyde by a ferredoxin‐dependent choline monooxygenase (CMO) (Brouquisse, Weigel, Rhodes, Yocum, & Hanson, 1989), and then, BADH converts betaine aldehyde to GB (Weretilnyk & Hanson, 1990).

The capability to accumulate GB varies among plants, with some genera classified as accumulators and some as nonaccumulators (Rhodes & Hanson, 1993). Several of the genes involved in GB biosynthesis have been isolated (Lamark et al., 1991; Rathinasabapathi et al., 1997; Weretilnyk & Hanson, 1990) and have been engineered into plants with the object to improve stress tolerance by increasing GB levels. Successful GB metabolic engineering has been reported for several plant species, mainly through overexpression of the bacterial or plant genes responsible for choline oxidation (Hayashi, Alia Mustardy, Deshnium, Ida, & Murata, 1997; Nuccio et al., 1998; Park et al., 2004; Sakamoto & Murata, 1998). Although the increase in GB in the reported studies was significant, accumulation was considerably lower than the GB level in high accumulator species. One possible explanation for this is that choline availability may limit GB accumulation in some plants. In fact, transgenic tobacco plants overexpressing CMO were able to accumulate large amounts of GB only when choline was supplemented (Nuccio et al., 1998).

*Zea mays* is considered a GB accumulator species, but certain genotypes are relatively deficient in GB. Brunk, Rich, and Rhodes (1989) demonstrated that maize genotypes could be generally classified into two distinct groups: low GB (L‐GB) and high GB (H‐GB) genotypes, with GB levels of less than 0.09 or more than 1.0 μmol/g fresh weight (FW), respectively. Rhodes and Rich (1988) reported that the L‐GB trait behaved as a single recessive gene, and Lerma et al. (1991) conducted complementation analysis to demonstrate that the L‐GB phenotype across several genotypes was attributable to a single locus, the recessive allele of what Yang et al. (1995) designated the *Bet1* gene. Although investigations of GB‐relevant metabolites and enzymes in near‐isogenic maize lines that differ at the *Bet1* locus have been conducted, the precise biochemical defect in L‐GB lines has not been determined (Peel, Mickelbart, & Rhodes, 2010; Yang et al., 1995).

We investigated further the genetic basis of the GB phenotype difference in maize to identify novel genes for GB engineering. Under the assumption that the GB difference in maize might be regulated at the gene transcription level, a gene expression analysis experiment was conducted to identify genes associated with the H‐GB phenotype. This work describes the discovery of a novel gene (*GB1*) that, when overexpressed, significantly increases GB accumulation in two tested species, maize and soybean.

## 2 MATERIALS AND METHODS

### 2.1 Gene expression analysis

For gene expression analysis, 98 inbred lines characterized for GB levels were used; of these, 59 were categorized as H‐GB and 36 as L‐GB. Ten replicates per genotype were planted and grown to the V7 stage of development. At V7, irrigation was withheld from half of the replicates to impose a drought condition from V8 to V10. For the irrigated section, irrigation was maintained during the entire course of the experiment. Leaf tissue was sampled from one plant per replicate when drought plots showed clear symptoms of dehydration stress (leaf rolling and leaf grayish cast). Total RNA was isolated from leaf tissue; cDNA for hybridization was synthesized from 1 μg of mRNA. The hybridization array used for analysis consisted of 5,749 elements, of which 4,433 represented unique genes.

### 2.2 Plant transformation

The *GB1* gene was amplified and cloned into a plant expression binary vector under control of the rice actin promoter or the 35S promoter, for maize and soybean, respectively. *Agrobacterium*‐mediated transformation of maize and soybean proprietary lines was carried out as previously described (Edgerton, Chomet, & Laccetti, 2003; Martinell et al., 2002).

### 2.3 *GB1* mapping

A B73xMo17 recombinant inbred line mapping population (Stuber, Lincoln, Wolff, Helentjaris, & Lander, 1992) was used to map the *GB1* locus in maize. RFLP markers *asg48* and *phi053*, defining the chromosome region 3.04 boundaries, were used to select recombinant inbred lines (RIL) homozygous for one of the parental alleles for both markers. Additional proprietary markers that map to the chromosome 3.04 region were used to confirm the absence of double recombination between the *asg48* and *phi053* markers. Two pools for each parental allele, each one composed of more than 15 individuals, were created (see supplemental information). To confirm that the individual pools were appropriate to map cDNA probes to the 3.04 chromosome region, the two RFLP probes *umc10* and *bnl15.20* were used as controls.

### 2.4 GB analysis

Approximately 30 mg FW of leaf tissue was collected from maize or soybean plants, freeze‐dried, and ground to fine powder. Powdered tissue was extracted with 80% ethanol, 0.1% formic acid, and 1 mM deuterated GB (d_9_gb) as a standard for 20 min and centrifuged to remove cell debris. Following filtration, GB content in the extract was determined by LC/MS‐MS analysis using an API 2000 system (Applied Biosystems, Foster City, CA, USA) equipped with an AllTech Alltima C18 column. GB concentration of the tissue sample was calculated as the GB/d_9_gb peak area ratio. For the genotype survey analysis, deuterated d_9_Val was added to the extraction buffer and the relative amount of GB was calculated as ratio with d_9_Val standard.

GenBank Accession Number: KU232555.

## 3 RESULTS

### 3.1 GB survey results categorize maize inbreds as H‐GB or L‐GB

**Figure 1:**
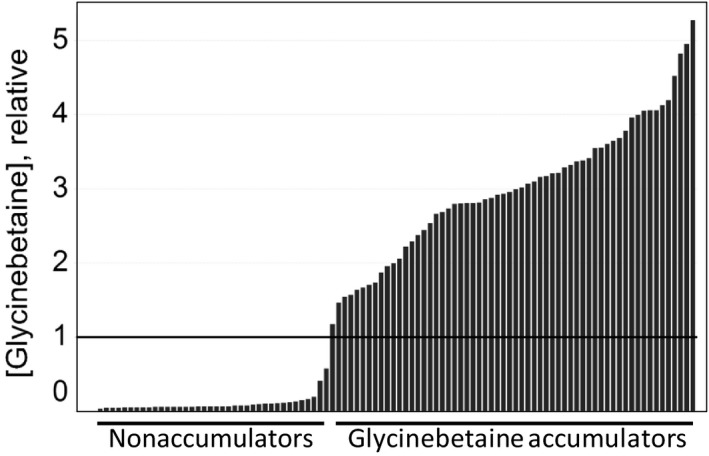
Maize genotypes divide into two GB phenotypic classes. Relative GB concentration in leaves for 98 genotypes used in the gene expression experiment is shown in rank order. Each bar represents the mean of 10 replicates, and the relative GB concentration was estimated as the ratio of GB peak area per internal standard peak area. One unit of [GB] is taken as the threshold distinguishing GB accumulators and nonaccumulators

Ninety‐eight inbred maize lines were surveyed for GB content, including proprietary lines, the widely used research lines Mo17 and B73, and 10 additional named inbreds that are available for noncommercial research purposes. (The latter may be requested through the USDA‐ARS Germplasm Resources Information Network [GRIN] National Plant Germplasm System [NPGS], using the inbred name to search by accession at the following link: http://www.ars-grin.gov/npgs/.) Leaf tissue samples of all lines were collected at the V10 (midvegetative) stage of development from both irrigated and drought‐stressed plants. Results of the GB survey confirmed the previously reported large genotypic variation for GB content in maize. As shown in Figure 1, GB content in the irrigated plants, determined as the relative amount of GB to an internal standard, ranged from 0.04 to 5.3. Most of the lines were unambiguously classified into one of two GB classes, with 59 genotypes classified as H‐GB and 36 as L‐GB lines. (Figure S1 displays glycinebetaine accumulation results for the 12 publicly available lines.)

#### 3.1.1 *GB1* mRNA is associated with GB accumulation

Having populated the L‐GB and H‐GB accumulating classes with a large number of maize lines, we reasoned that, if the expression level of a small number of genes was primarily responsible for the difference in plant GB content, it might be possible to identify those genes by a comparative transcript array analysis. A DNA array containing 5,749 elements, for a total of 4,433 unique gene sequences, was developed to evaluate relative abundance of mRNAs isolated from leaves of the 98 survey inbreds. Gene elements were selected for the array based on three factors: putative stress responsiveness, association with the GB pathway, or putative involvement in osmotic adjustment pathways. We first considered if the genotype average signal, with no treatment distinction, could determine if any gene was differentially expressed between H‐GB and L‐GB lines, based on average signal intensities for each array element between the two classes. Eleven genes showed significant statistical difference (*p* < .001) in relative mRNA abundance, with six genes being induced in the H‐GB and five induced in the L‐GB class.

**Figure 2:**
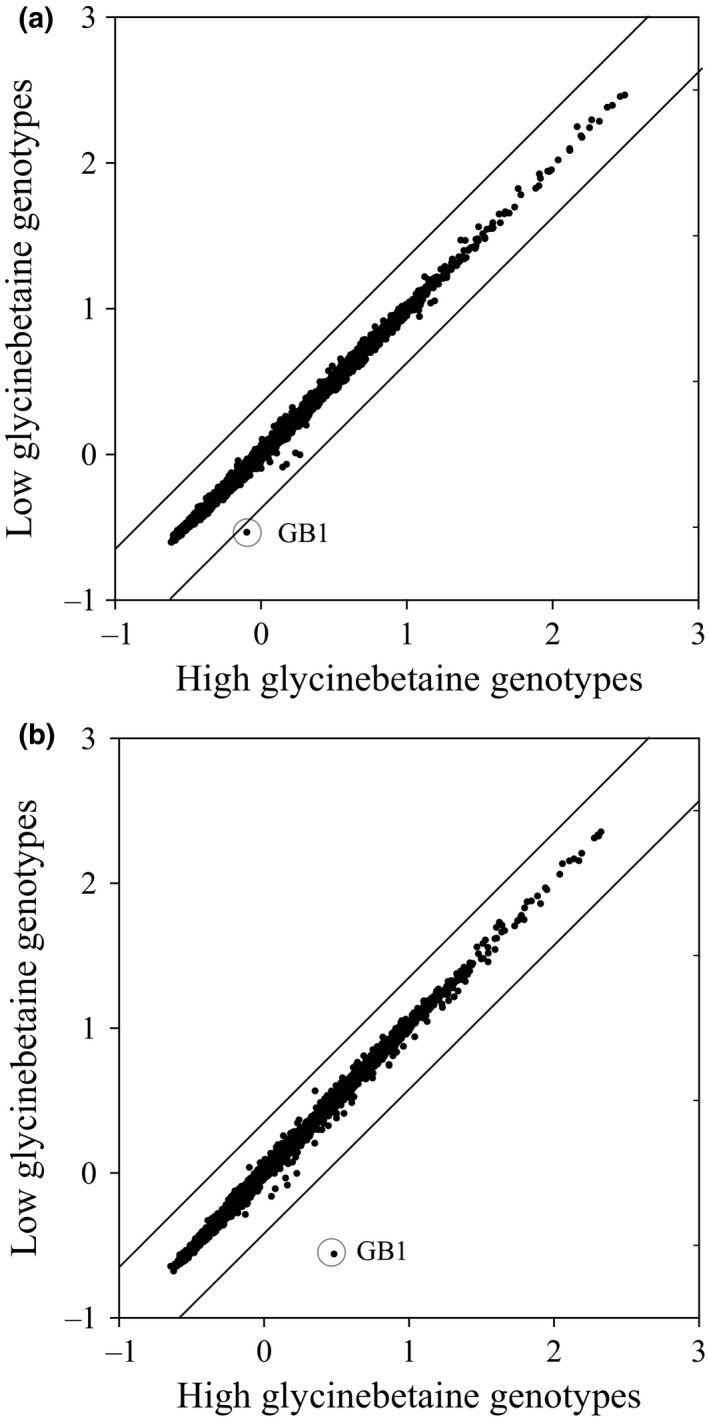
The *GB1* gene is differentially expressed in H‐GB maize genotypes. Each panel reports the difference in gene expression between high and low GB genotypes for irrigated (a) and drought (b) conditions. Log‐transformed array element hybridization signal intensity of each replication was normalized by subtracting the median of the noncontrol elements. Average signal values were calculated for high and low GB genotype classes and the difference between classes is presented in the scatter plot. The diagonal lines delimit the twofold difference in gene expression between the two GB classes. The differential signal for the *GB1* element is circled

Of those eleven genes, only one, described here as *GB1* (GenBank Accession KU232555), had a greater than twofold change in abundance between the H‐GB and L‐GB well‐watered plants (Figure 2) Under drought conditions, the difference in *GB1* mRNA levels between the H‐GB and L‐GB classes was more pronounced (Figure 2).

Putative proteins with high similarity to the *GB1* gene product have been identified from various genome sequencing projects including maize (NP_001136959), sorghum (XP_002455692), and *Setaria italica* (XP_012702021). The predicted GB1 protein shows 60% identity to a rice putative C‐4 sterol methyl oxidase (GenBank AAN62786) and 36% identity to the functionally characterized Arabidopsis protein SMO1 (Genbank AAQ13424), an ortholog of the yeast ERG25 gene involved in sterol biosynthesis. However, these enzymes are thought to be involved in sterol metabolism and no role has been identified for these enzymes in the synthesis of GB. The deduced amino acid sequence indicates GB1 is a member of the Pfam fatty acid hydroxylase superfamily with the signature motif between amino acids 130 to 243. Bioinformatic prediction of subcellular location by MultiLoc2 (Blum, Briesemeister, & Kohlbacher, 2009) suggests a peroxisomal location, although this has not been experimentally verified.

#### 3.1.2 *GB1* transcription is induced by drought in H‐GB lines

**Figure 3:**
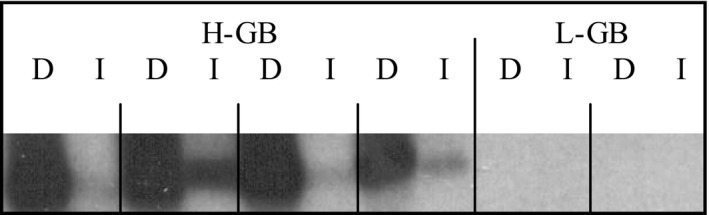
*GB1* expression is induced by drought. Blot analysis of RNA isolated from H‐GB and L‐GB lines under drought (D) and irrigated (I) conditions, using *GB1* cDNA as probe

We selected four representative H‐GB lines based on the magnitude of their high *GB1* expression and compared these to two L‐GB lines to confirm the array results. RNA blot analysis showed *GB1* mRNA accumulation under both irrigated and drought conditions for the H‐GB but not for the L‐GB lines (Figure 3). For the H‐GB lines, *GB1* mRNA accumulation was substantially higher in plants subjected to drought stress.

To demonstrate that the higher *GB1* expression in H‐GB genotypes was not simply the result of the higher GB levels, we tested if exogenously provided GB had any effect on *GB1* mRNA accumulation. Plants from one H‐GB genotype and one L‐GB genotype were grown in a greenhouse and irrigated with a 50 mM GB solution starting 6 weeks after planting. GB analysis of plant tissue revealed that the compound was rapidly absorbed and transported to the leaves, but RNA blot analysis showed that the added GB did not increase *GB1* transcript accumulation (Figure S2).

### 3.2 *GB1* maps to the *bet1* locus region

Cosegregation of the *GB1* with the previously mapped *bet1* locus (Rhodes et al., 1993) was investigated to establish the relationship between the sequence reported here and the previously reported major determinant for GB accumulation in maize. A recombinant inbred line (RIL) mapping population developed by a cross of two H‐GB lines was used for the analysis. As described in the Section 2, two pools of individuals were created to map cDNA probes to the maize 3.04 genome region. The Southern blot hybridization patterns of the selected RIL pools were compared to the parental and F1 generation. Two RFLP probes, *umc10* and *bnl15.20*, mapped inside and outside, respectively, the 3.04 chromosome region boundaries and confirmed that the constituted pools were suitable to establish if cDNA probes were in tight linkage with the selected genome region. The probe *umc10* shows a hybridization pattern characteristic for a linked probe. RFLP analysis using *GB1* cDNA as probe revealed the hybridization pattern expected for probes located in the 3.04 chromosome region (see Figure S2), suggesting that *bet1* and *GB1* may be tightly linked.

No significant or relevant differences between nonaccumulators and accumulators have been identified in gene sequences near the *GB1* coding region. However, this analysis is incomplete; while high‐resolution sequence data have been gathered for some maize inbreds, the GB status of these has not been characterized. B73 and Mo17, two inbreds with extensive genomic information available, are both H‐GB lines (see Figure S1).

### 3.3 *GB1* overexpression increased GB in maize and soybean

Transgenic maize plants constitutively overexpressing *GB1* were generated by *Agrobacterium tumefaciens* transformation. Plants regenerated from in vitro culture were assayed at the V6‐V8 developmental stage for leaf GB content. Average GB content for the GB1 transgenic lines was 7.1 mM, compared to 0.1 mM GB for the transformation genotype, a L‐GB line. Although biological replication was not possible due to only one R0 plant per transgenic line, we could detect GB concentrations up to 20 mM for a few transgenic lines, corresponding to a more than 200‐fold increase. The leaf GB content of the transgenic plants was notably higher than that observed in nontransgenic H‐GB maize growing in the same environment.

**Figure 4:**
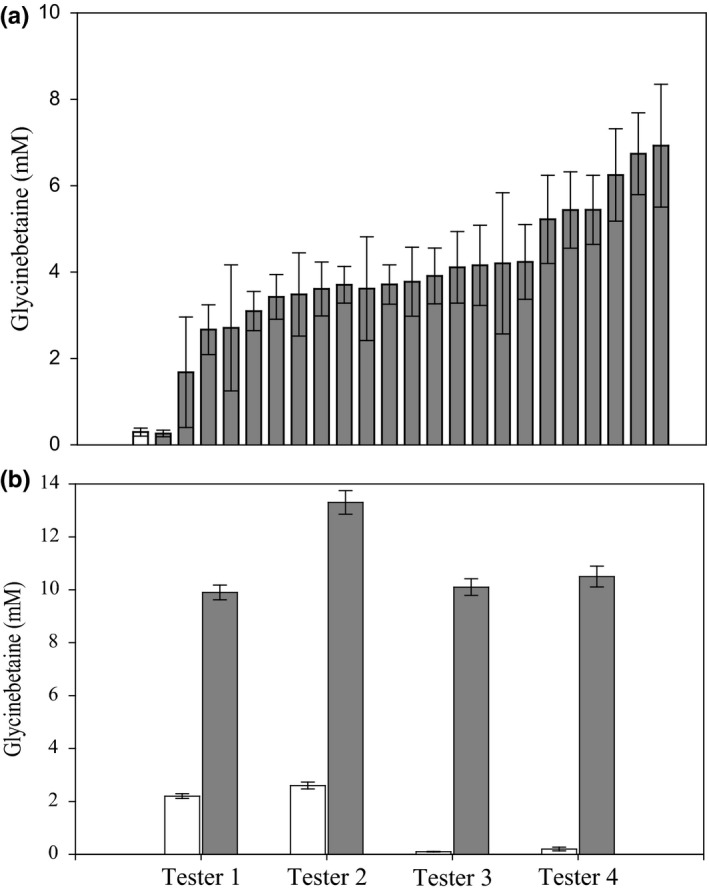
*GB1* transgenic expression increases GB in maize. Each bar represents the mean GB concentration ± standard error. (a) Mean GB concentration in V5 leaves of 23 maize GB1 transgenic hybrid events (gray bars) compared to control (white bar). Each bar represents the average of eight replicates. (b) Mean GB concentrations in VT‐stage leaves of GB1 transgenic plants for hybrids generated using four different testers, two H‐GB and two L‐GB. For each testcross, data from 22 (for Testers 1 and 3) or eight (for Testers 2 and 4) independent transgenic lines were pooled. The white bars indicate controls; the gray bars indicate transgenic events

To confirm that *GB1* overexpression effectively increased GB in maize, 23 independently regenerated lines were selected for analysis. Four different hybrid sets were developed by crossing each GB1 transgenic line to two H‐GB and two L‐GB genotypes. Plants of each of the four hybrids were grown in the field and assayed for GB at both early (V5) and late (VT) vegetative stages. Most transgenic plants overexpressing the *GB1* gene accumulated substantially more GB than control plants at both assayed stages. For example, for one H‐GB tester, 22 of the 23 GB1 lines assayed at V5 showed substantial GB increases compared to the negative control (Figure 4). Notable GB accumulation persisted in transgenic hybrids with both H‐GB and L‐GB testers throughout development, as revealed by the assessment of leaf tissue samples collected at the VT‐stage (Figure 4).

**Figure 5:**
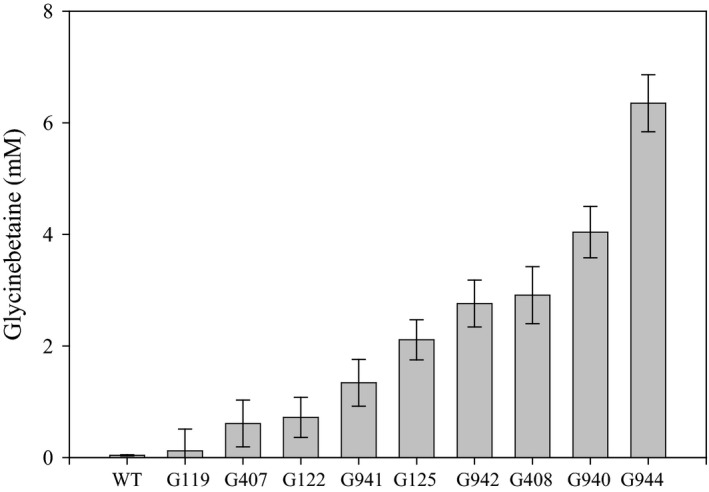
*GB1* transgenic expression increases GB in soybean. GB concentration in leaves of soybean *GB1* transgenic events compared to wild‐type control (WT) 6 weeks after planting. Each bar represents the average of six replicates ± standard error

To investigate whether *GB1* expression could affect GB accumulation in other crop plant species, we also generated GB1 transgenic soybean lines. Nine soybean GB1 transgenic lines were assayed for GB leaf content 6 weeks after planting, and eight of the lines showed a substantial GB increase (Figure 5) relative to the nontransgenic control.

## 4 DISCUSSION

GB enhancement through metabolic engineering has been associated with an improvement in plant stress tolerance, but efficacy of this engineering in a field environment for important crops has not been demonstrated. One explanation for this could be the limited GB increases previously achieved through genetic engineering. We rationalized that factors other than the known GB metabolic enzymes could play a critical role for GB accumulation in maize. We thus decided to seek such additional factors by investigating the differences in gene expression for genotypes with opposite GB phenotypes to gain additional knowledge relative to the genetic control of GB accumulation in maize.

Our high‐throughput GB assay enabled the survey of many genotypes. The unambiguous classification of the genotypes into the correct GB class was critical to design a powerful experiment to assess differential gene expression (Figure 1). The large number of genotypes, coupled with the reliable GB classification, minimized the variability in gene expression not related to GB content, as demonstrated by the extremely low number of genes differentially expressed. Interestingly, only the *GB1* gene was exceptionally differentiated when all genotypes were considered for the analysis (Figure 2). Our data demonstrate that *GB1* expression is not dependent on the GB concentration and that constitutive overexpression of the gene is sufficient to effectively increase GB content in both maize and soybean. The amount of GB accumulated in the maize and soybean GB1 transgenic plants indicates that overexpression of *GB1* overcomes barriers to GB accumulation in these species. Our data, the mapping of *GB1* locus in the same 3.04 chromosome region as *bet1*, and the demonstration that *GB1* transgenic expression can result in substantial accumulation of GB even in a L‐GB genetic background are consistent with the hypothesis that GB1 is the *Bet1* gene product.

Interestingly, the GB1 protein sequence does not contain any domain characteristic of proteins known to be involved in GB synthesis, such as choline oxidase. As a member of the fatty acid hydroxylase superfamily and given that choline is a key substrate for GB synthesis, it is possible that GB1, either alone or in combination with other enzymes, allows the phosphatidylcholine (PC) pool to be used as a precursor of choline. Building on prior analyses indicating that large amounts of PC are present in the plastidial outer membrane envelope, Dorne, Joyard, Block, and Douce (1985) found that PC is the major polar lipid of the outer leaflet of the chloroplast outer envelope membrane and therefore accessible from the cytosolic side of the membrane. Given that GB1 has putative transmembrane domains, but not organelle targeting signals, it is possible that GB1 is inserted from the cytosol to the outer organellar surface.

Nuccio et al. (2000) built on evidence that cytosolic choline is precursor to GB synthesized in the chloroplast. Modeling established that choline availability is a factor that limits the synthesis of GB in tobacco expressing a chloroplastic GB pathway, suggesting that subcellular choline localization influences the overall accumulation of GB. It may be possible that choline released via a GB1‐associated pathway is tied to choline transport into the chloroplast. This hypothesis suggests that the phosphatidylcholine pool or flux through the outer leaflet should be different between maize GB nonaccumulators and accumulators.

Our preliminary experiments showed that some of the GB1 transgenic lines developed negative visual phenotypes. It is probable that constitutive overexpression may negatively impact untargeted cellular functions.

We describe here a novel gene that plays a pivotal role controlling GB accumulation in plants. We have demonstrated that *GB1* can be used to increase GB concentration in plants through overexpression technologies such as transgenesis. Optimized expression of *GB1* may offer an innovative tool to control GB accumulation in plants as a strategy to improve tolerance to abiotic stress and enhance and stabilize yield for crop species.

## AUTHOR CONTRIBUTIONS

R.J.B. conceived the original screening and research plans; P.C. designed experiments; P.C., A.L., B.S.H., E.B., D.E.N., R.J.B., A.F.R, and M.G. performed experiments and interpreted data; S.B. provided critical information on maize inbreds; P.C., D.E.N., and E.B. wrote the article with contributions of all authors.
