# Supplementary material for: Identification of GB1, a gene whose constitutive overexpression increases glycinebetaine content in maize and soybean
Source: Plant Direct. 2018 Feb 22;2(2):e00040. doi: 10.1002/pld3.40 (PMC6508499; doi:10.1002/pld3.40)
Supplement: Supplementary file 1 [file PLD3-2-e00040-s001.pdf]

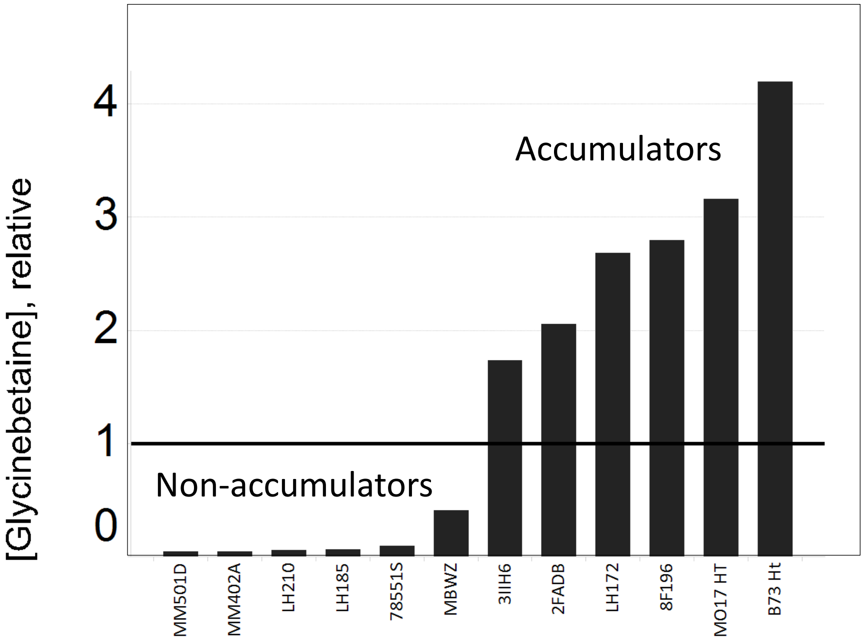

Figure S1. Twelve publicly available lines, including 6 accumulators and 6 non-accumulators, have a GB accumulation pattern similar to the entire set of germplasm studied.

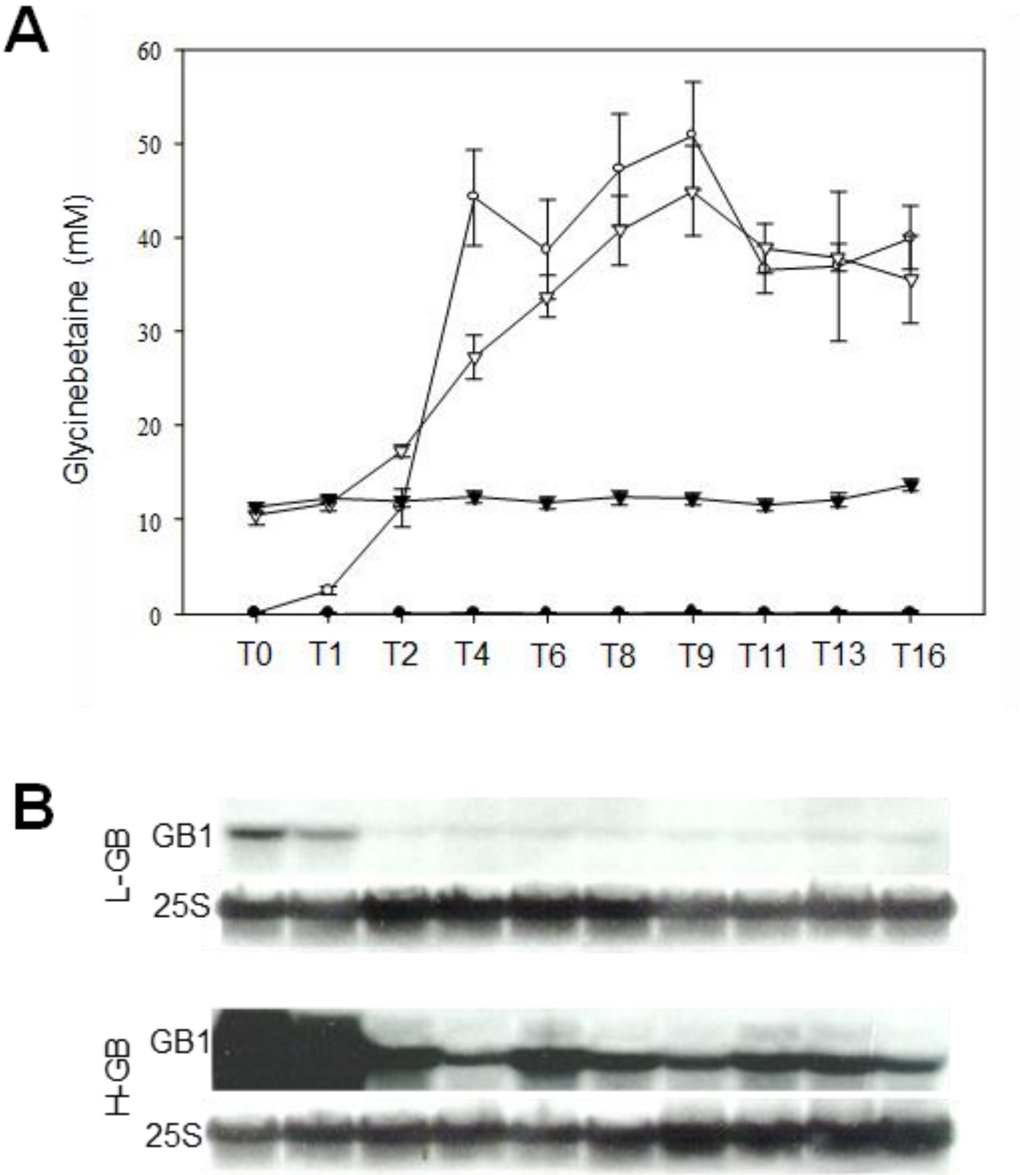

Figure S2. GB1 mRNA accumulation is not induced by exogenous GB.

A. GB concentration in leaves of H-GB and L-GB genotypes irrigated with a GB solution. Average of three replicates is indicated for H-GB (triangles) and L-GB (circles) genotypes for both 50 mM GB (open symbols) and water control treatments (filled symbols). T0 samples were harvested at treatment start, T1 3 hours after first GB irrigation, T2 to T16 indicate number of days from T0.
B. Northern blot analysis for H-GB and L-GB genotypes irrigated with GB. GB1 cDNA and 25S rRNA cDNA were used as probes. Lanes correspond with sample times indicated in panel A.

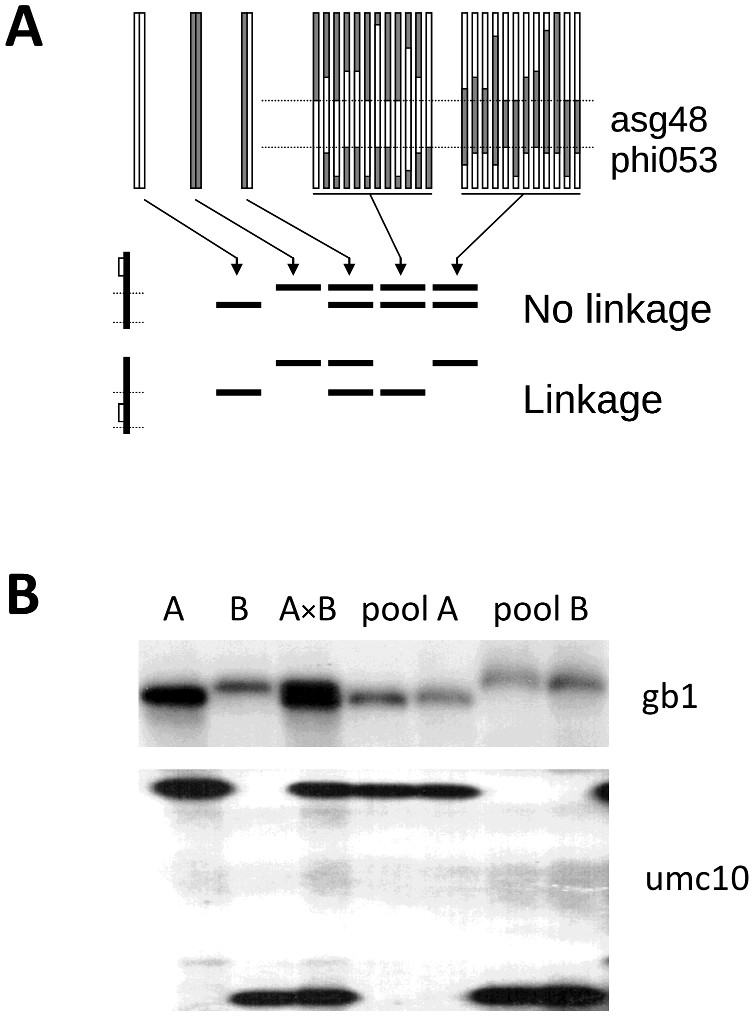

Figure S3. *GB1* maps to the 3.04 chromosome region. (A) Schematic diagram of the RIL selection used for *GB1* mapping and expected hybridization pattern for linked and not linked DNA fragments. (B) Southern blot analysis of B73×Mo17 RIL mapping population using gb1 or umc10 as probes. A = B73 parental line; B = Mo17 parental line; A×B = F1 cross; Pool A = RILs homozygous for allele B73 at 3.04 region; Pool B = RILs homozygous for allele Mo17 at 3.04 region.
